# R-YOLO: A YOLO-Based Method for Arbitrary-Oriented Target Detection in High-Resolution Remote Sensing Images

**DOI:** 10.3390/s22155716

**Published:** 2022-07-30

**Authors:** Yongjie Hou, Gang Shi, Yingxiang Zhao, Fan Wang, Xian Jiang, Rujun Zhuang, Yunfei Mei, Xinjiang Ma

**Affiliations:** 1 College of Information Science and Engineering, Xinjiang University, Urumqi 830017, China; 18434753056@stu.xju.edu.cn (Y.H.); zhaoyingxiang@stu.xju.edu.cn (Y.Z.); xmilu0315@stu.xju.edu.cn (F.W.); jxian1996@stu.xju.edu.cn (X.J.); zhuangrj@stu.xju.edu.cn (R.Z.); cb2@stu.xju.edu.cn (Y.M.); 2 Geomatics School of Earth Sciences and Engineering, Hohai University, Nanjing 211100, China; mxj@hhu.edu.cn

**Keywords:** deep learning, remote sensing image, rotating frame, attention mechanism, YOLOv5

## Abstract

In view of the existence of remote sensing images with large variations in spatial resolution, small and dense objects, and the inability to determine the direction of motion, all these components make object detection from remote sensing images very challenging. In this paper, we propose a single-stage detection network based on YOLOv5. This method introduces the MS Transformer module at the end of the feature extraction network of the original network to enhance the feature extraction capability of the network model and integrates the Convolutional Block Attention Model (CBAM) to find the attention area in dense scenes. In addition, the YOLOv5 target detection network is improved by incorporating a rotation angle approach from the a priori frame design and the bounding box regression formulation to make it suitable for rotating frame-based detection scenarios. Finally, the weighted combination of the two difficult sample mining methods is used to improve the focal loss function, so as to improve the detection accuracy. The average accuracy of the test results of the improved algorithm on the DOTA data set is 77.01%, which is higher than the previous detection algorithm. Compared with the average detection accuracy of YOLOv5, the average detection accuracy is improved by 8.83%. The experimental results show that the algorithm has higher detection accuracy than other algorithms in remote sensing scenes.

## 1. Introduction

With the rapid development of space remote sensing technology, high-quality remote sensing images have increased, and target detection has made great progress in remote sensing images. Remote sensing image target detection is used to find the specific location of the target of interest in the remote sensing image, and identify its target category, usually with special attention to aircraft, airports, cars, bridges and other targets. This technology plays an important role in the civil and military fields, such as port and airport flow monitoring, traffic diversion, looking for lost ships and so on, providing new solutions for national defense, maritime, natural resource management and other fields, further improving resource utilization, and strengthening national defense early warning capabilities.

Common deep learning target detection algorithms are divided into two categories: single-stage detection algorithms and two-stage detection algorithms. The single-stage detection algorithm adopts the idea of target regression and only needs to perform feature extraction once to detect the target, such as YOLO [1,2,3,4], and SSD [5]. The single-stage detection algorithm is fast and has low accuracy. The detection performance is poor, and it can easily miss detection. The two-stage detection algorithm first generates candidate regions through special modules, classifies the candidate regions, and finally determines the detection results by the Non-Maximum Suppression method [6], such as R-CNN [7], Fast R-CNN [8], and Faster R-CNN [9]. The two-stage detection algorithm is slow but has high accuracy.

Object detection algorithms based on remote sensing images have been a research hotspot in recent years. Wang et al. [10] proposed an end-to-end multi-scale visual attention network (MS-VANs) method in 2019, by using a hopping encoder model to extract multi-scale features from full-scale images, learning visual attention network and classification and regression branches on each single-scale feature map, and finally using a hybrid loss function to train the model to solve the complex background problem in remote sensing images; Zhang et al. [11] proposed a Hierarchical robust CNN. The spatial semantic information is represented by the extracted multi-scale convolution features, and the fully connected layer features are superimposed to further cope with the scale change problem and improve the robustness problem caused by image scaling and rotation, but the detection speed is not good; Li et al. [12] proposed a remote sensing target detection algorithm framework, including a local context feature fusion network and a region proposal network (RPN). The dual-channel feature network combines the path learning local and context attributes in the processing layer. On the basis of scale, multi-angle anchors are added to adapt to multi-view problems; Pang et al. [13] proposed the Tiny-Net lightweight residual structure, extracting features from the input process and each patch uses a classifier to verify the existence. The network adds classifiers and detectors to each other in an end-to-end fashion, which can further speed up the processing. Cheng et al. [14] combined the SSD algorithm to improve the RPN network and introduced a feature fusion layer to improve the detection performance of small objects. Liu et al. [15] proposed using the anchor-free algorithm CenterNet, with the flexibility of a detector to detect remote sensing images, which further proved that the CenterNet algorithm had better performance in detection accuracy and speed than the anchored YOLOv3 algorithm but also had some small and dense targets missed and wrongly detected. Qu et al. [16] proposed introducing a convolutional block attention module and adaptive feature fusion method in the YOLOv3 network model to improve the detection speed and accuracy of remote sensing image targets. Adam et al. [17] proposed the YOLT algorithm by adjusting the output layer of the feature map, cropping the image, and using the convolutional neural network to detect objects of different scales, which is suitable for object detection of remote sensing images with large resolution. However, the detection of remote sensing image targets is still very challenging because it has a series of questions such as complex backgrounds, large changes in spatial resolution, small and many objects to be detected, irregular arrangement, and is easily disturbed by external factors such as weather and so on.

The contributions of our study are as follows: A dense target detection method based on YOLOv5 is proposed. In order to better match image features, the new boundary discontinuous free rotation detector is used to solve the angle periodicity problem by transforming the angle regression problem into a classification problem, and evaluating the angle error after classification by using the long-side representation method. At the same time, the feature network of the original network is improved. A Swin Transformer [18] module is used to replace the original terminal full convolution operation, so as to strengthen the feature extraction ability of the network model. At the same time, in order to find the attention region in the specific large coverage image, we use the convolution block attention module. In addition, the detection performance is improved by balancing the focusing loss and combining the LRM [19] method, and the remote sensing image detection method is further optimized.

## 2. Methodology

### 2.1. Background of YOLOv5

**Figure 1:**
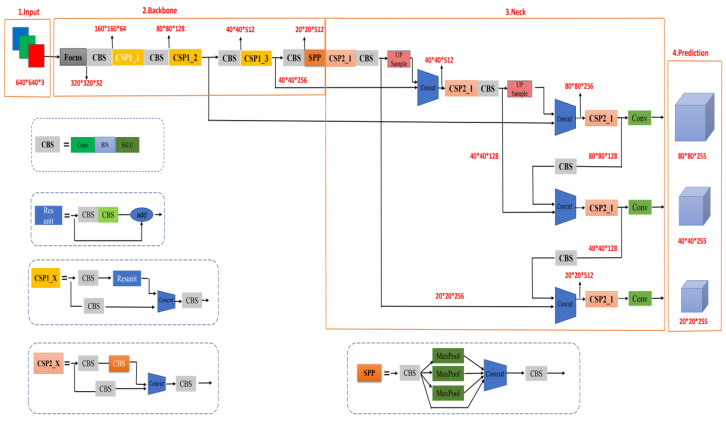
YOLOv5s network structure. It is mainly composed of three parts: backbone network, neck network, and prediction network.

YOLOv5 is a single-stage target detection algorithm. According to the size of the model, YOLOv5 has four versions, namely, YOLOv5s, YOLOv5m, YOLOv5l and YOLOv5x. The weight, width and depth of the four versions increase successively. The network model is mainly divided into four parts: input end, backbone end, neck end and output end. Its network structure is shown in Figure 1.

The input of YOLOv5 consists of three parts: mosaic data enhancement, adaptive anchor box calculation and adaptive image scaling. Among them, Mosaic data enhancement includes three methods: random scaling, random cropping, and random arrangement. Since the trigger probability of Mosaic can be random, the image splicing position and picture can be randomly selected, and other enhancement operations can be used to increase the number of images of a training. In addition, it can effectively prevent over-fitting and further enhance the robustness of the model. The splicing feature of Mosaic can greatly improve the uneven distribution of targets in the DOTA data set, and due to the randomness of its splicing, as the training time increases, the improvement effect will be more obvious. The Mosaic data enhancement method uses 4 pictures, which are spliced according to random scaling, random cropping and random arrangement. This enhancement method can combine several pictures into one, which can not only enrich the data set, but also greatly improve the training speed of the network, and reduce the memory requirements of the model. In the YOLO series of algorithms, for different data sets, it is necessary to set anchor boxes of specific length and width. In the network training phase, the model outputs the corresponding prediction frame based on the initial anchor frame, calculates the gap between it and the GT frame, and performs a reverse update operation to update the parameters of the entire network. YOLOv5 will run a separate program to get the initial anchor box function and embed it into the code. At each training time, the optimal anchor box is adaptively calculated according to the name of the data set. Adaptive image scaling is to scale the image to a uniform size. A method proposed by YOLOv5 can adaptively add the least black borders to the zoomed image, which further improves the inference speed of the YOLOv5 algorithm.

**Figure 2:**
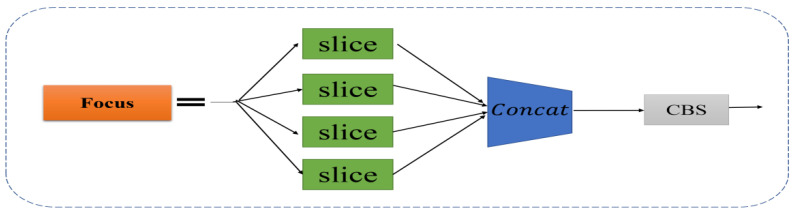
Focus network structure. The structure is mainly to slice the feature into four parts, and then perform the Concat in the channel dimension.

The backbone network of YOLOv5 is mainly composed of the Focus module, improved CSP structure, SPP and other modules. The Focus structure mainly cuts the input image through the Slice operation. For example, the original input image size is 608 × 608 × 3. After Slice and Concat operations, a 304×304×12 feature map is output; then a 304×304×32 feature map is output through a Convolutional layer with 32 channels. By reducing the number of operating parameters of Focus, the network speed is further improved. The structure of the Focus module is shown in Figure 2. The CSP structure consists of several bottleneck modules. The bottleneck is a classic residual structure. After the input passes through two convolutional layers, the Add operation is performed with the original value to complete the residual feature transfer without increasing the output depth. The improved CSP module sets up two CSP structures in the backbone network and uses the CSP1-X residual structure for feature extraction to further optimize the gradient information and reduce the amount of calculation. The SPP module adopting the spatial pyramid idea further expands the receptive field to enrich the feature map expression ability, and finally converts the input of different sizes to the output of the same size, which solves the problem of inconsistent input images.

The Neck network still uses the FPN [20] +PAN [21] structure, which further improves the feature fusion ability of the network. FPN adopts a downsampling operation. PAN adopts an upper operation. YOLOv5 draws on the CSP2 structure designed by CSPNet. It combines the conventional FPN layer with the bottom-up feature pyramid and fuses the extracted semantic features and position features. At the same time, the feature fusion of the backbone layer and the detection layer enables the model to obtain richer feature information.

In YOLOv5, CIOU_Loss is used as the loss function of the Bounding box. Head outputs a vector with the class probability of the target object, the object score, and the location of the bounding box for that object. The detection network consists of three detection layers, and feature maps of different sizes are used to detect target objects of different sizes. Each detection layer outputs the corresponding vector, and finally generates the predicted bounding box and category of the object in the original image and labels it.

### 2.2. Improved Network Structure

**Figure 3:**
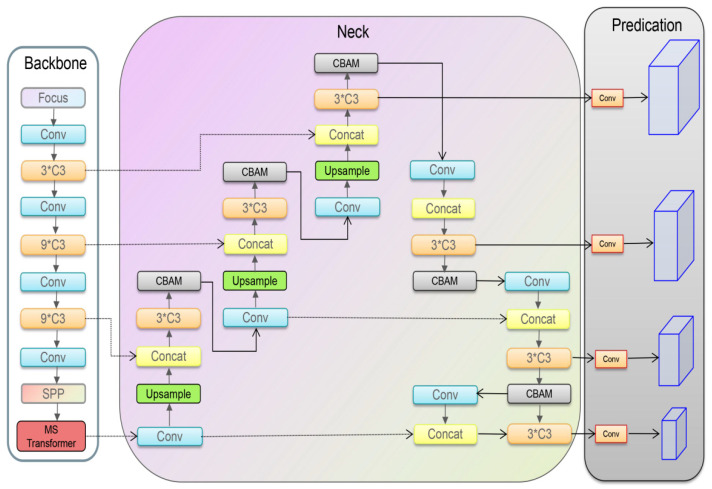
Network structure diagram of improved YOLOv5. The overall network structure consists of three parts: backbone network, feature fusion network and output layer.

This paper is optimized based on the YOLOv5s network, and the improved structure is shown in Figure 3. The overall network structure consists of three parts: backbone network, feature fusion network and output layer. Among them, the backbone network aggregates different images with fine granularity and forms a convolutional neural network of image features. By introducing the MS Transformer block at the end of the backbone network, the c3mstr module is used to replace the original full convolution operation to enhance the model’s performance of the global awareness with lower overhead. The attention mechanism is integrated into the feature fusion network, and the spatial attention and channel attention modules are given the same priority to enhance the expression effect of the image in a weighted manner. Finally, the output layer will replace the original loss function based on the focal loss function to solve the problem of computational loss caused by sample imbalance.

## 3. Proposed Method

In remote sensing scenes, due to the perspective problem, the objects in the image are usually small in scale and clustered, unlike the large and salient objects in natural images, which makes it impossible to extract detailed information about the objects by using the feature extraction network in natural scenes. If the network level is deepened, the limited details of the small target itself will be lost. This module draws on the idea of a Swin Transformer and proposes an MS Transformer backbone network. By adopting a window-based multi-attention mechanism, these operations can further reduce the computational power in the case of low-resolution feature maps, so that the feature extraction module can better capture global information and rich contextual information.

**Figure 4:**
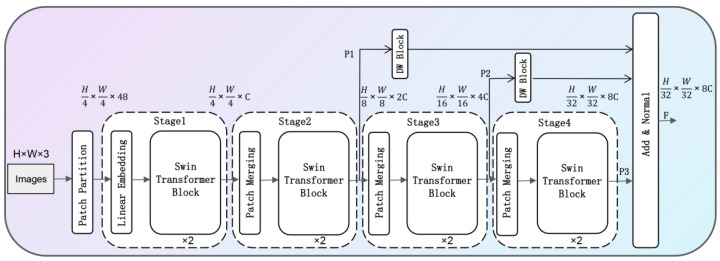
MS Transformer module structure.

The MS Transformer network architecture is shown in Figure 4 below. The downsampling operation for feature extraction of the input image includes four processing stages. First, the input RGB image is divided into a small non-overlapping feature map block Partition module, and the features are extracted from them according to the sequence elements to model the semantic information globally. After the Linear Embedding operation, the block diagram is mapped to a 96-dimensional space, and the semantic feature information is obtained by successively using two consecutive layers of a Swin Transformer Block. At the end of each stage, the dimension of the input is reduced through the process of Patch Merging to further improve the receptive field of the patch and window, and make the hierarchical feature representation better. After four processing procedures, four feature maps with different multiples are obtained. In this paper, the dimension of the feature maps of downsampling ratios are unified by the DW Block and then the feature maps are normalized to obtain the output feature map.

**Figure 5:**
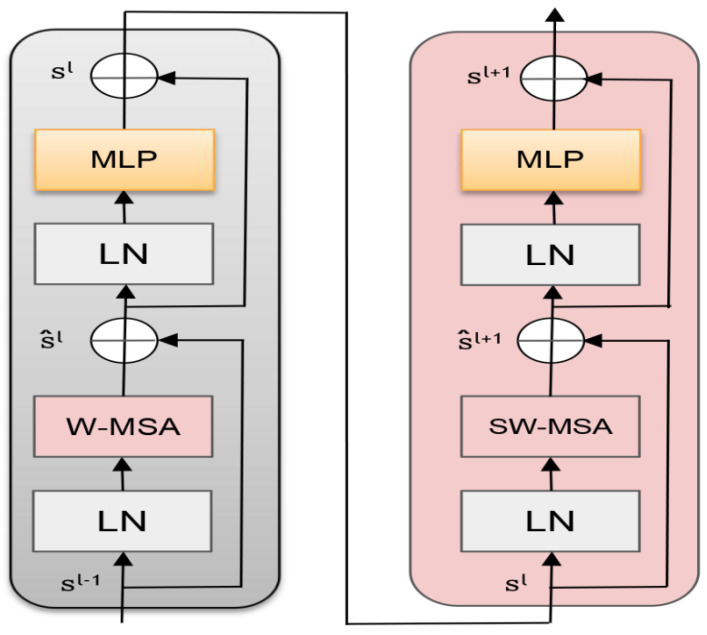
Two successive Swin Transformer Blocks.

The structure of the Swin Transformer Block is shown in Figure 5 below, which mainly includes Layer Norm LN, Window-based Multi-head Self-Attention (W-MSA), and Shifted Window Multi-head Self-Attention (Shifted). Window-based Multi-head Self-Attention, SW-MSA) and the multilayer perceptron are constructed by residual connections. The structure mainly uses the Window Muti-head Self-Attention module (W-MSA) to take the local window as the unit, ensure the image features and reduce the computational complexity by paying attention to the window. Then, the Shift Window Multi-Head Self-Attention Module (SW-MSA) is used to translate the feature map in different directions according to half the window length to realize the interaction between cross-window features and enhance the feature map extraction ability. 

The overall calculation formula of the Swin Transformer module is as follows:

(1) $${\hat{S}}^{l}=W-MSA\left(LN\left(S^{l-1}\right)\right)+S^{l-1}$$

(2) $$S^{l}=MLP\left(LN\left({\hat{S}}^{l}\right)\right)+{\hat{S}}^{l}$$

(3) $${\hat{S}}^{l+1}=SW-MSA\left(LN\left(S^{l}\right)\right)+S^{l}$$

(4) $$S^{l+1}=MLP\left(LN\left({\hat{S}}^{l+1}\right)\right)+{\hat{S}}^{l+1}$$

In the formula, $S^{l}$ and ${\hat{S}}^{l}$ represent the output results of MLP and self-attention in the first block, respectively, $S^{l-1}$ is the input image sequence, $S^{l+1}$ is the output image feature sequence, LN is the layer normalization process, W−MSA is the window multi-head self-attention module, and MLP is Multi-layer perceptron. SW−MSA is a shifted window multi-head self-attention module.

**Figure 6:**
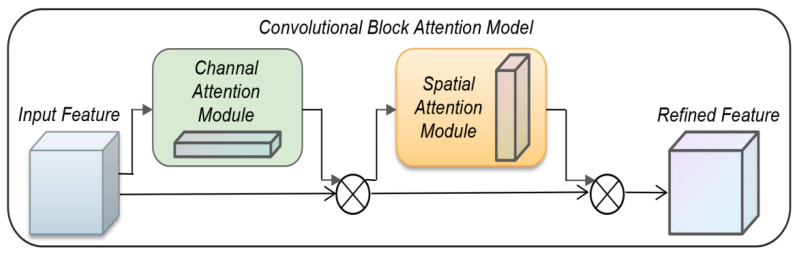
CBAM structure diagram.

A deep convolutional neural network can extract hierarchical features from images, in which the low-level network outputs simple geometric features and the high-level network outputs abstract semantic features. Convolutional Block Attention Model (CBAM) [22] is a simple and effective attention module for a feedforward convolutional neural network. Given an intermediate feature map, the CBAM module will sequentially infer the attentional map along two independent dimensions (channel and space), and then multiply the attentional map by the input feature map for adaptive feature optimization. The output results of the convolution layer will first pass through a channel attention module to get weighted results and then pass through a spatial attention module to get final weighted results, as shown in Figure 6. In view of the complex background of remote sensing images in this paper, which affects the feature extraction effect of detection targets, especially the problem that features of low-resolution feature images will be lost after convolution operation, this problem can be solved well by adding a convolution attention mechanism into residual blocks.

The calculation formulas of the channel attention and spatial attention modules are:

(5) $$M_{C}(F)=\sigma (MLP(AvgPool(F)+MLP(MaxPool(F)))$$

(6) $$M_{S}(F)=\sigma (f^{7\times 7}(Concat(AvgPool(F),MaxPool(F))))$$

In the formula, F is the input feature map, $M_{C}(F)$ is the channel attention module, σ is the sigmoid function, MLP is the multi-layer perceptron of the fully connected layer and the ReLU activation function. AvgPool is the average pooling function, and MaxPool is the maximum pooling function. Function. f is a 7 × 7 convolution operation. Based on Formulas (5) and (6), the output formula after introducing the residual network is shown in Formulas (7) and (8):

(7) $$F_{C}=M_{C}(F)⊗F$$

(8) $$F_{S}=M_{S}(F)⊗F$$

In the formula: $F_{c}$ represents the channel attention output feature map, $M_{c}$ represents the channel attention module operation, F represents the input feature map, ⊗ represents element-by-element multiplication, and $F_{s}$ represents the spatial attention output feature map. The principle is to learn the feature map space and channel information of the previous layer to obtain different weights, obtain the channel attention through the channel attention mechanism, and then multiply the weights with the original feature map, respectively, to obtain the feature map after the attention mechanism is merged and achieve the integration of goals. By introducing the convolution block attention model, the saliency of the target in the image can be improved, and the feature expression ability of the target in the detection network can be enhanced. Therefore, the detection accuracy of the target can be improved.

In the evaluation of target detection performance, the problem of unbalanced foreground and background samples will always be encountered. Especially in the data set of this paper, the background occupies a large proportion, and the proportion of the foreground is too small. Most of the bounding boxes are matched by the bounding boxes marked as background class boxes. At the same time, the number of samples of some categories is very small, and it is easy to be dominated by other categories in the data so that the information provided by a small number of samples cannot play a normal role in the loss function, and ultimately it is difficult to provide a loss function that correctly guides model training. The original YOLOv5 version uses horizontal bounding boxes, and the loss function is divided into three parts, confidence loss, class classification loss, and box bounding box regression loss. In the algorithm of this paper, the rotation angle information is introduced to adjust the horizontal frame detection to the rotation frame detection. The loss part consists of four parts: confidence loss, class classification loss, frame regression loss, and θ angle classification loss. In order to avoid the boundary discontinuity problem caused by the calculation of the BECWithLogitsLoss function, the long-edge labeling method, CSL [23], of the circular smooth label is used, and the final loss output is shown in Formula (9):

(9) $$L_{\mathrm{total}}=L_{CIOU}+L_{obj}+L_{cls}+L_{angel}$$

Focal Loss [24] has the function of adjusting sample imbalance and mining samples (samples that are difficult to be learned). By increasing the weight of cross entropy Loss, it solves the problems of positive and negative sample distribution and imbalance between simple samples and complex samples. Cross Entropy (CE) is a commonly used loss measure function. The formula is shown in Equation (10). p represents the probability corresponding to the model prediction category, and y is the sample category label, with a value of 1 or 0.

(10) $$CE(p,y)=\left\{\begin{array}{l} -log_{a}p,y=1\\ -log_{a}(1-p),y=0 \end{array}\right.$$

Using the balance factor α in the cross-entropy loss can solve the problem of the uneven proportion of positive and negative samples in the data set. By making the coefficients of a small number of positive samples larger and the coefficients of a large number of negative samples smaller, the model can learn more useful features. At the same time, a hyperparameter adjustment factor y is introduced to make the loss function pay more attention to complex and difficult samples. The formula for calculating Focal Loss is as follows:

(11) $$FL(p,y)=\left\{\begin{array}{l} -{\left(1-p\right)}^{y}log_{a}p,y=1\\ -p^{y}log_{a}(1-p),y=0 \end{array}\right.$$

Combining Focal Loss with the balance factor, the final Focal Loss formula can be obtained, and the calculation formula is as follows:

(12) $$FL(p,y)=\left\{\begin{array}{l} -a{\left(1-p\right)}^{y}log_{a}p,y=1\\ -(1-a)p^{y}log_{a}(1-p),y=0 \end{array}\right.$$

Due to the influence of the hyperparameter regulating factor γ, the bounding frame loss is ignored. To solve this defect, the hyperparameter factor ξ is added to the algorithm in this paper, which has a higher proportion in the overall Loss function and improves the performance of the overall network in calculating Focal Loss. Meanwhile, in order to reduce the influence of foreground–background samples in the YOLOv5 real-time detector, LRM filters out the predicted low loss values before backpropagation, leaving only the high loss values that are favorable to the detector.

**Figure 7:**
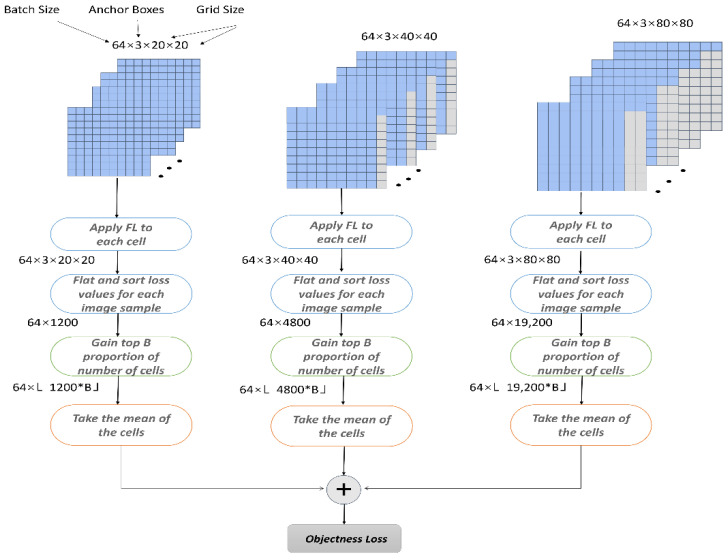
Comprehensive loss function calculation diagram.

This paper is further modified on the basis of LRM. By selecting ranking factors for each feature graph, it is removed with inappropriate values in backpropagation, and the most appropriate loss value is selected as the detection value. For example, if *b* = 0.3 and the feature graph size is 3 × 20 × 20, the 360 cells with the highest detection loss are selected, and the other remaining cells are excluded from the backpropagation process. Finally, by combining the two difficult sample mining methods, the target detection performance is further improved, as shown in Figure 7.

## 4. Experiments

### 4.1. Data Set

The DOTA [25] data set is an image data set used for aerial remote sensing in object detection, with a total of 2806 aerial images and a total of 188,282 manually annotated instances. The resolutions of each image range from 800 × 800 to 4000 × 4000 and the data set contains objects of different scales, orientations and shapes. There are 15 categories of labeled instances: airplanes, ships, storage tanks, baseball fields, tennis courts, basketball courts, ground tracks, ports, bridges, large vehicles, small vehicles, helicopters, roundabouts, soccer fields, and basketball courts. There are horizontal object frame (HBB) and directed target frame (OBB) versions of the annotation file. This paper selects the directed data annotation method of the DOTA data set and converts it to long-edge representation for training and prediction. Because the size of the DOTA data set is too large, the input of the ordinary detection network will be too slow to calculate. In this paper, the image will be cropped before the input network, and a sub-image of 1024 × 1024 will be obtained. In order to avoid the problem of not losing edge information, the rate of reserved overlapping area will be set at 20%. The processed training set contains 15,749 images, and the validation set contains 5297 images.

**Figure 8:**
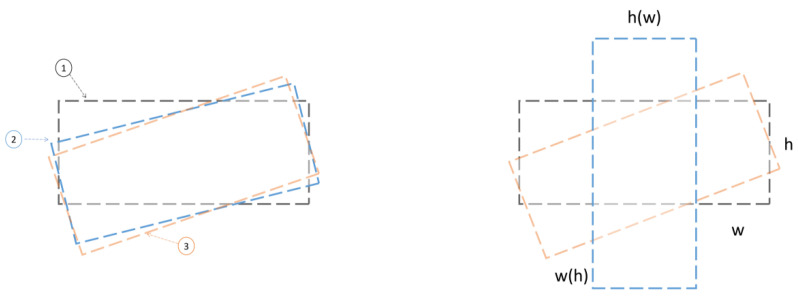
PoA and EoE problem diagram.

Aiming at the diversity of the existing scale of the detection target, the target with a large length and width is very sensitive to the change of angle, and the horizontal bounding box is used to obtain the orientation information of the movement of the target object. In this paper, the rotation frame annotation method is adopted. Common arbitrary rotation frame methods include opencv representation and long edge representation. Opencv mainly contains five parameters [*x, y, w, h,* *θ*], where *θ* is the acute angle between the rotating coordinate system and the x-axis, and the counterclockwise direction is specified as a negative angle, so the angle range is [−90°, 0). As shown on the left of Figure 8, in the target rotation detection, the ideal regression method is from the black box to the red box counterclockwise, but due to the periodicity of angles (PoA) problem, box ① is rotated 3° counterclockwise to become box ②, and box ① is rotated through 87° to reach the location of box ③. Therefore, the losses caused by the resulting periodic jump problem caused are great. As shown in the figure on the right, if you rotate clockwise from the black box to the red box, you need to scale w and h at the same time, which causes the exchange of edges (EOE) to a certain extent and increases the difficulty of regression. In this paper, the long-side representation method is adopted. In the long-side definition method based on 180 degrees, only the angle θ has a boundary problem in the process from the black frame to the red frame, and the influence of the loss value only comes from the angle periodicity problem (PoA).

**Figure 9:**
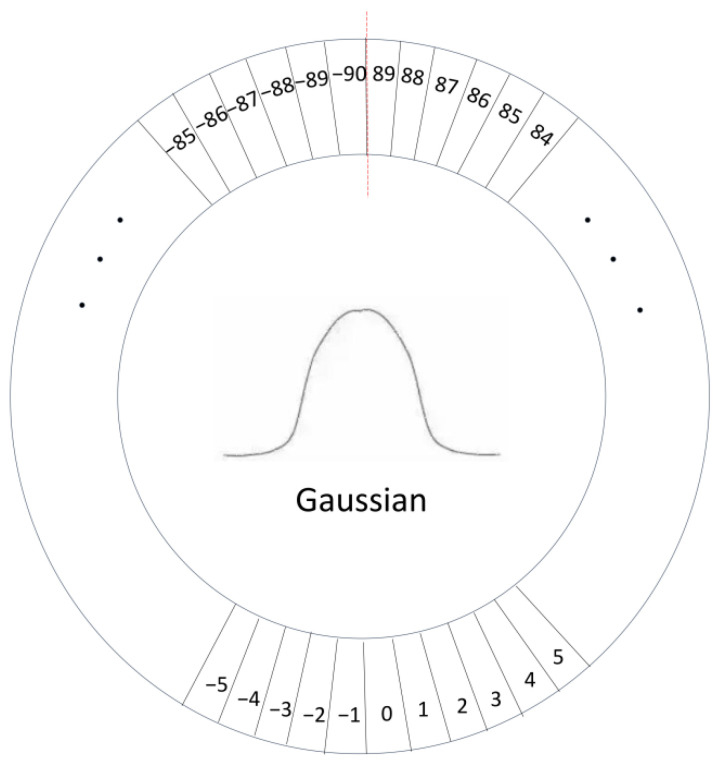
Circle smooth label diagram.

For a series of problems such as a sudden increase in loss value and increased difficulty in network learning caused by the sudden increase in angle difference and boundary difference, the method of angle regression is converted into a classification form, and classification can be carried out to avoid the definition that exceeds the angle range. When converting to a simple classification problem, it is inevitable that there will be a loss of accuracy, such as in the case of a first-class case, where it is impossible to predict the degree of the decimal point. Moreover, the long-side representation requires the form of five parameters, and the angle range is between 180 degrees, otherwise, there will be a problem with variable interactivity. Based on the above problems, circular smooth labels (CSL) are introduced to avoid edge cases. This paper also uses the Gaussian function as the window function, as shown in Figure 9 below. Through the setting of the window function, the angular distance between the predicted label and the ground truth label is measured, and within a certain range, the predicted value loss corresponding to the detection value closer to the true value is smaller.

The expression of the ring smooth label is as follows:

(13) $$CSL(x)=\left\{\begin{array}{l} g(x),\theta -r<x<\theta +r\\ 0,otherwise \end{array}\right.$$

(14) $$g(x)=ae^{-{\left(x-b\right)}^{2}/2c^{2}}$$

In the formula, g(x) is the window function, and r controls the window radius. In this paper, the Gaussian function is selected as the window function, which can make the obtained label reflect the angle distance between each θ, and can also give the label function the same period according to the periodicity of angle θ, so that the time difference value at the calculation boundary is very large and the loss is very small. The window radius, r is 6, and the specific expression is:

(15) $$CSL(x)=\left\{\begin{array}{l} ae^{-{\left(x-b\right)}^{2}/2c^{2}},\theta -6<x<\theta +6\\ 0,otherwise \end{array}\right.$$

In the formula, a, b, and c are real constants, and a > 0. θ is the value of the rotation angle.

### 4.2. Evaluation Methods

In order to evaluate the performance of the network and validate the effectiveness of the network model, this paper adopts the mean average precision (mAP) as the evaluation index in object detection. In order to calculate mAP, we first need to calculate the precision rate (*Precision*) and the recall rate (*Recall*) indicators. The accuracy rate is the ratio of the number of predicted positive samples to all positive samples, and its calculation formula is as follows:

(16) $$P=\frac{TP}{TP+FP}\times 100\%$$

In the formula, P represents the precision rate, which represents the ratio of the correct samples to the total number of samples in the detection process. FP represents the number of falsely detected samples in the detection process, that is, the number of samples that are not real targets but detected, TP represents the number of correctly detected samples in the detection process, that is, the number of samples detected by the real target, the recall rate is used to represent the ratio of the number of positive samples detected in the network, and its calculation formula is as follows:

(17) $$R=\frac{TP}{TP+FN}\times 100\%$$

In the formula, R represents recall rate, FN represents the number of correct samples predicted to be wrong samples in the detection process, that is, the number of negative samples incorrectly classified. To synthesize the model performance, the accurate recall curve was used to show the balance between detectors.

Average accuracy AP refers to the curve enclosed by the recall rate. The recall is the horizontal axis and the precision rate is the vertical axis, and the area under the curve is the AP value. Its calculation formula is as follows:

(18) $$AP=\int_{0}^{1}P\mathrm{dR}$$

The average accuracy rate mAP (Mean Average Precision) is used as a measure of the algorithm performance of the model. The formula for calculating mAP is:

(19) $$mAP=\frac{1}{C}\sum_{c=1}^{c}AP_{C}$$

### 4.3. Experimental Results and Analysis

**Figure 10:**
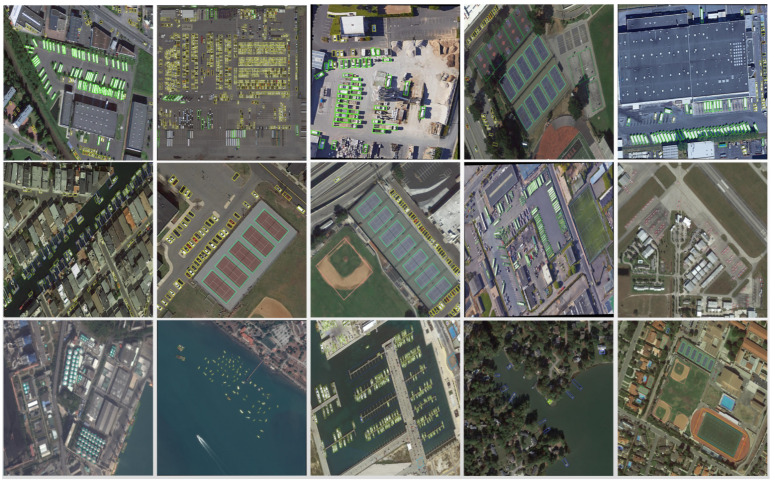
Visualization results of DOTA image detection.

Part of the detection effect of the algorithm in this paper is shown in Figure 10. It can be seen that the prediction frame is more accurate in the detection of densely arranged ships, large vehicles, storage tank scales, etc., which further reduces the impact of the background on the target classification. Secondly, the target information of aircraft, vehicles, ships and so on, containing the direction of movement is further obtained; at the same time, the overlapping problem caused by the horizontal boundary prediction frame for dense targets is reduced. For the detection of remote sensing images with obvious scale changes, the detection performance is further improved.

At the same time, in order to further verify the performance of the algorithm in this paper, this paper compares and analyzes the current popular remote sensing image target detection algorithms, and tests the accuracy on the validation set. The comparison algorithms include the FR-O based on Faster R-CNN [25] algorithm, the RIO [26] algorithm for two-stage detection with a priori box, the IENet [27] algorithm without a priori box, the $R^{2}$CNN [28] algorithm based on Faster R-CNN and the $R^{3}$Det [29] for refining rotating object detectors algorithm, the RCNet [30] algorithm, the SCRDet [31] algorithm with better robustness to small, cluttered and rotating objects, the RSDet [32] function for rotating object loss, using the segmentation method for rotating object detection, the Mask OBB [33] algorithm and the YOLOv5 algorithm of the horizontal box. The experimental results are shown in Table 1.

It can be seen from Table 1 that the overall effect of the algorithm in this paper is better than that of the current popular detection algorithms in the detection of DOTA remote sensing image targets. Although it is inferior to other methods in the detection of some target categories, such as the ground runway category, which is detected best in the RIO algorithm, and the basketball court and helicopter categories, which are detected better in the Det algorithm. However, it is very effective for the modules added in this paper in the categories of strong density. Especially when detecting target categories such as airplanes, ships, tennis courts, large vehicles, and storage tanks, the detection accuracy of the algorithm in this paper is 90.15%, 90.15%, 89.32%, 90.84%, 86.97%, and 89.06%, which are 0.84 percentage points, 10.7 percentage points, 1.08 percentage points, 12.94 percentage points, and 7.7 percentage points higher than the original YOLOv5 algorithm, respectively. The average accuracy of the improved YOLOv5 algorithm proposed in this paper is higher than other algorithms, and its mAP reaches 77.01%, which is 8.83% higher than the original YOLOv5 algorithm. In general, it shows that the algorithm in this paper has relatively few false detections and missed detections, which verifies the effectiveness of the added module for remote sensing dense image detection.

$R^{2}$ $R^{3}$

### 4.4. Ablation Experiments

In order to fully verify the effectiveness of the module improvement in this paper, experimental analysis is performed on the DOTA data set to verify the importance of each proposed component. Each component is sequentially embedded into the YOLOv5s model, and the same training techniques and environmental conditions are used in each set of experiments. The results are shown in Table 2.

In the above table, improvement 1 means using the long-side representation to realize the rotation of the target frame, and using the classification idea to replace the influence of periodic changes caused by the regression problem on network training; improvement 2 means adding the MS Transformer module based on a Swin Transformer in the backbone network; improvement 3 means adding an attention mechanism to the Neck layer; improvement 4 is an improvement on the loss function, combining two difficult sample mining methods to reduce the loss value. When these four module improvement points are added to the model, the evaluation accuracy value is increased by 8.83 percentage points, which greatly improves the detection effects of remote sensing images with high density and small targets.

## 5. Conclusions

Aiming at the problems that remote sensing images have densely arranged targets, small targets and complex backgrounds, and the common algorithms of natural scenes cannot meet the detection requirements, this paper proposes an improved YOLOv5 remote sensing image target detection method. The algorithm uses a rotating frame to deal with the problem that the horizontal bounding box struggles to obtain the orientation information of the target object’s motion. At the same time, the ring label method is used to reduce the influence of the change of loss value for the periodic problem caused by angle regression. The global perception capability of the model is enhanced by incorporating an MS Transformer into the feature extraction network, while the convolutional block attention model, CBAM, is integrated to find attention regions in dense scenes. Finally, a combination of two difficult sample mining methods is used to improve the focal loss function. The experiments show that the detection accuracy of the improved algorithm model is further improved, and the improved Focal Loss further reduces the impact of the loss value caused by the bounding box and foreground–background samples. This paper has generally achieved good recognition results on the DOTA data set, but the improvement of the detection accuracy of helicopters, bridges and other categories is still very low. Mainly due to factors such as illumination, occlusion, complex background, and low image resolution, the next step will be to optimize and improve the model to further improve the detection effect while ensuring speed.

## Figures and Tables

**Table 1:** Comparison of experimental results of different methods for each category. Expression for each category in the table: PL for aircraft, BD for baseball field, BR for bridge, GTF for track and field, SV for small car, LV for large car, SH for boat, TC for tennis court, BC for basketball court, ST for oil storage tank, SBF for football field, RA for circular lane, HA for port, SP for swimming pool, HC for helicopter.

Methods	PL	BD	BR	GTF	SV	LV	SH	TC	BC	ST	SBF	RA	HA	SP	HC	mAP
FR-O	79.42	77.13	17.70	64.05	35.30	38.02	37.16	89.41	69.64	59.28	50.30	52.91	47.89	47.40	46.30	54.13
IENet	80.20	64.54	39.82	32.07	49.71	65.01	52.58	81.45	44.66	78.51	46.54	56.73	64.40	64.24	36.75	57.14
$R^{2}$CNN	80.94	65.67	35.34	67.44	59.92	50.91	55.81	90.67	66.92	72.39	55.06	52.23	55.14	53.35	48.22	60.67
RRPN	88.52	71.20	31.66	59.30	51.85	56.19	57.25	90.81	72.84	67.38	56.69	52.84	53.08	51.94	53.58	61.01
RCNet	88.12	73.10	35.15	55.01	54.75	71.65	76.12	90.63	67.55	76.11	48.43	61.29	63.18	62.34	15.31	62.58
RIO	88.53	77.91	37.63	**74.08**	66.53	62.97	66.57	90.50	79.46	76.75	59.04	56.73	62.54	61.29	55.56	67.74
SCRDet	89.98	80.65	52.06	68.36	68.36	60.32	72.41	**90.85**	**87.94**	86.86	65.02	66.68	66.25	68.24	65.21	72.61
$R^{3}$Det	89.49	81.17	50.53	66.10	70.92	78.66	78.21	90.81	85.26	84.23	61.81	63.77	68.16	69.83	**67.17**	73.74
RSDet	90.1	82.0	53.8	68.5	70.2	78.7	73.6	91.2	87.1	84.7	64.3	68.2	66.1	69.3	63.7	74.1
MaskOBB	89.56	**85.95**	54.21	72.90	76.52	74.16	85.63	89.85	83.81	86.48	54.89	**69.64**	73.94	69.06	63.32	75.33
YOLOv5	89.31	76.38	47.33	61.21	71.32	74.03	78.62	89.76	82.23	81.36	60.93	63.88	65.24	68.36	60.13	68.18
Ours	**90.15**	84.51	**54.27**	68.45	**78.86**	**86.97**	**89.32**	90.84	74.26	**89.06**	**66.78**	67.84	**74.54**	**74.21**	65.13	**77.01**

**Table 2:** Experimental comparisons of each combination in the feature extraction network.

Method	MS Transformer	CBAM	CSL	Loss	mAP/%
YOLOv5					68.18
1			√		74.04
2	√		√		75.53
3	√	√	√		76.72
4	√	√	√	√	77.01

## Data Availability

Not applicable.
